# National Trends in Physical Activity Among Adults in South Korea Before and During the COVID-19 Pandemic, 2009-2021

**DOI:** 10.1001/jamanetworkopen.2023.16930

**Published:** 2023-06-05

**Authors:** Sangil Park, Hyeon Jin Kim, Sunyoung Kim, Sang Youl Rhee, Ho Geol Woo, Hyunjung Lim, Wonyoung Cho, Dong Keon Yon

**Affiliations:** 1 Department of Neurology, Kyung Hee University Hospital, Kyung Hee University College of Medicine, Seoul, South Korea; 2 Center for Digital Health, Medical Science Research Institute, Kyung Hee University Medical Center, Kyung Hee University College of Medicine, Seoul, South Korea; 3 Department of Regulatory Science, Kyung Hee University, Seoul, South Korea; 4 Department of Family Medicine, Kyung Hee University Medical Center, Kyung Hee University College of Medicine, Seoul, South Korea; 5 Department of Medical Nutrition, Graduate School of East-West Medical Science, Kyung Hee University, Yongin, South Korea; 6 Department of Endocrinology and Metabolism, Kyung Hee University College of Medicine, Seoul, South Korea; 7 Department of Pediatrics, Kyung Hee University Medical Center, Kyung Hee University College of Medicine, Seoul, South Korea

## Abstract

**Importance:**

There is a lack of comprehensive data on the association of the COVID-19 pandemic with the prevalence of physical activity in large-scale data sets.

**Objective:**

To investigate long-term trends in physical activity using information from a nationally representative survey covering 2009 to 2021.

**Design, Setting, and Participants:**

This general population–based and repeated cross-sectional study was conducted from 2009 to 2021 using the Korea Community Health Survey, a nationally representative survey in South Korea. Data from 2009 to 2021 for 2 748 585 Korean adults were obtained through a nationwide, large-scale, serial study. Data were analyzed from December 2022 through January 2023.

**Exposure:**

COVID-19 pandemic onset.

**Main Outcomes and Measures:**

The trend of sufficient aerobic physical activity was measured by prevalence and mean metabolic equivalent of task (MET) score based on World Health Organization physical activity guidelines and defined as 600 MET-min/wk or greater. The cross-sectional survey included age, sex, body mass index (BMI), region of residence, education level, income level, smoking status, alcohol consumption level, stress status, physical activity level, and history of diabetes, hypertension, and depression.

**Results:**

Among 2 748 585 Korean adults (738 934 aged 50-64 years [29.1%] and 657 560 aged ≥65 years [25.9%]; 1 178 869 males [46.4%]), the prevalence of sufficient physical activity did not change significantly during the prepandemic period (β difference, 1.0; 95% CI, 0.6 to 1.4). During the pandemic, the prevalence of sufficient physical activity decreased significantly, from 36.0% (95% CI, 35.9% to 36.1%) in 2017 to 2019 to 30.0% (95% CI, 29.8% to 30.2%) in 2020 and 29.7% (95% CI, 29.5% to 29.9%) in 2021. Trends showed decreases in the prevalence of sufficient physical activity among older adults (ages ≥65 years; β difference, −16.4; 95% CI, −17.5 to −15.3) and younger adults (ages 19 to 29 years; β difference, −16.6; 95% CI, −18.1 to −15.0) during the pandemic. In particular, the trend of sufficient physical activity declined during the pandemic in females (β difference, −16.8; 95% CI, −17.6 to −16.0), individuals in urban residences (β difference, −21.2; 95% CI, −22.2 to −20.2), healthy participants (eg, those with normal BMI, 18.5 to 22.9: β difference,−12.5; 95% CI, −13.4 to −11.7), and individuals at increased risk of stress (eg, history of a depressive episode; β difference, −13.7; 95% CI, −19.1 to −8.4). Prevalence trends in mean MET score were similar to those in the main results; total mean MET score decreased from the 2017 to 2019 period (1579.1 MET-min/wk; 95% CI, 1567.5 to 1590.7 MET-min/wk) to the 2020 to 2021 period (1191.9 MET-min/wk; 95% CI, 1182.4 to 1201.4 MET-min/wk.

**Conclusions and Relevance:**

This cross-sectional study found that the national prevalence of physical activity was stable or consistent before the pandemic period, with a marked decrease during the pandemic, particularly among healthy individuals and subgroups at increased risk of negative outcomes, including older adults, females, urban residents, and those with depressive episodes. Future studies may be needed to evaluate the association between the COVID-19 pandemic and changes in physical activity.

## Key Points

**Question:**

How did physical activity change among adults in Korea from 2009 to 2021, a period that included the COVID-19 pandemic?

**Findings:**

This cross-sectional study of 2 748 585 Korean adults found that the prevalence of physical activity was stable or consistent before the COVID-19 pandemic period, with a marked decrease in physical activity during the pandemic. Subgroups at increased risk, including older adults, females, urban residents, and those with depressive episodes, experienced a significant decline in physical activity levels, as did healthy individuals.

**Meaning:**

This study found that the prevalence of physical activity was stable or consistent before the pandemic but declined during the pandemic in 2020 and 2021.

## Teaser

This cross-sectional study investigates changes in physical activity among adults in South Korea from before to during the COVID-19 pandemic.

## Introduction

The World Health Organization (WHO) defines physical activity as any bodily movement produced by skeletal muscles that requires energy expenditure; this includes activities undertaken while working, playing, carrying out household chores, traveling, and engaging in recreational pursuits.^1,2^ According to WHO 2020 guidelines,^3^ all adults should engage in moderate- or vigorous-intensity physical activity each week or an equal balance of moderate- and vigorous-intensity aerobic physical activity. Individual factors and the social environment have been found to be associated with the adoption of physical activity,^4^ despite its potential association with reduced public health care expenses. A 2016 study^5^ found that decreased physical activity was associated with as much as $53.8 billion in costs in the international global health system

The emergence of the highly contagious SARS-CoV-2 virus in the COVID-19 pandemic and responding containment efforts were certainly associated with changes in individuals’ lifestyles.^1,2^ Strict social distancing regulations, which resulted in the closure of gymnasiums, swimming pools, and sports facilities, were associated with changes in physical activity.^6^ People worldwide reported difficulties in participating in physical activity, with prevalence decreasing by up to 50% during the pandemic.^7^ However, there is a lack of comprehensive data demonstrating the association of the COVID-19 pandemic with the prevalence of physical activity in large-scale data sets.^8^

This study aimed to examine physical activity trends over 13 years using nationally representative data collected from 2009 to 2021. Using a sample size of more than 2 million adult participants, we investigated changes in the prevalence of physical activity among Korean adults from before the COVID-19 pandemic to during the pandemic.

## Methods

The protocol for this cross-sectional study was approved by the Korea Disease Control Agency (KDCA) and University of Sejong . Written voluntary consent was obtained from all participants during the enrollment period from 2009 to 2021. This study followed the Strengthening the Reporting of Observational Studies in Epidemiology (STROBE) reporting guideline.

### Study Design and Population

In this study, large-scale representative data were collected from 2009 to 2021 by the Korea Community Health Survey (KCHS), which was conducted by the KDCA. The survey collected information on health behaviors, body measurements, and health-related outcomes from adult members (aged ≥19 years) of sample households through interviews. Sampling was conducted to ensure the probability of proportional sampling by considering the number of households by housing type; a secondary sample household was selected for systematic sampling. From a total of 220 000 people per year, 900 people were surveyed in each region. Some adults may have completed the same survey more than once.

This study included 2 748 585 adult participants who completed the survey between 2009 and 2021. We disregarded responses from participants who did not provide information on their region, body mass index (BMI; calculated as weight in kilograms divided by height in meters squared), or physical activity; as a result, the final sample size was reduced to a total of 2 543 652 adult participants of the study.

### Covariate Definitions

We considered 12 covariates concerning participant characteristics: age, sex, region of residence (urban and rural),^9^ BMI (underweight, normal weight, overweight, and obese), educational level (≤high school or ≥college), income level (quartile 1 [lowest], quartile 2, quartile 3, and quartile 4 [highest]), smoking status (current smoker, former smoker, and never smoked), alcohol intake level (0, 1-4, and ≥5 d/wk), previous history of diabetes or hypertension, a depressive experience within 1 year of the survey, and receipt of mental health counseling owing to stress.^10,11^ Following the WHO Asia-Pacific guidelines, BMI was divided into 4 groups: underweight (<18.5), normal (18.5-22.9), overweight (23.0-24.9), and obese (≥25).^12^ Diabetes and hypertension were defined by whether a practicing physician had diagnosed the participant with the condition. The KCHS does not provide data on participant race or ethnicity given that this information was not collected.

### End Points

According to WHO physical activity guidelines, adults should aim to achieve at least 150 minutes of moderate-intensity or 75 minutes of vigorous-intensity aerobic physical activity per week or some equivalent combination of moderate-intensity and vigorous-intensity aerobic physical activity.^3^ This is equivalent to 600 metabolic equivalent of task (MET)-min/wk.

The total MET score was calculated as follows: Vigorous MET-minute/week = 8.0 × vigorous-intensity activity minutes × vigorous-intensity days. Moderate MET minutes/week = 4.0 × moderate-intensity activity minutes × moderate-intensity days. Total physical activity MET minutes/week = vigorous + moderate MET minutes/week. The level of physical activity was classified into 2 groups according to the MET score: greater than and less than the recommended level of physical activity.

### Statistical Analysis

Statistical analyses were conducted using SAS statistical software version 9.4 (SAS Institute); Python 3 programming language version 3.10.4 (Python Software Foundation), with software libraries Pandas version 1.5.0, SciPy version 1.9.2, Statsmodels version 0.13.2, and NumPy version 1.23.3; and Excel version 2021 (Microsoft). Consecutive years of data were merged and filtered according to this study’s criteria.^13^

We analyzed KCHS data from 2009 to 2021 to calculate the national trend in the prevalence of physical activity and the mean MET score. To determine the trend in prevalence of physical activity, we divided the period into 3 sections: prepandemic (2009-2019), early pandemic (2020), and midpandemic (2021). In the prepandemic category, years were combined into sets of 2 years to stabilize each estimate.^12^ The KCHS did not have a physical activity–related investigation in 2018; therefore, we excluded the 2018 KCHS data set. The main purpose of this study was to investigate the association of COVID-19 with physical activity based on trends in physical activity rates over the past 13 years.

The level of physical activity was classified into 2 groups: more than (sufficient aerobic physical activity) and less than (insufficient aerobic physical activity) the recommended amount of physical activity based on a MET score of 600 points. For trends of prevalence and mean MET scores before and during the COVID-19 pandemic, we obtained β coefficients with 95% CIs using a generalized linear model. We further analyzed β differences to characterize the differences between prepandemic and pandemic periods. A logistic regression model was used to calculate odds ratios (ORs) with 95% CIs of physical activity from 2020 to 2021 vs those from 2017 to 2019.^14,15^ Additionally, we conducted linear, quadratic, and cubic spline interpolations to examine linear and nonlinear trends in physical activity and changes in prevalence; we used a heat map to visualize local shifts. All estimated values were significant, with a 2-sided *P* value with a significance level of .05. If 95% CIs did not include 1 for ORs or 0 for β values, they were considered statistically significant (equivalent to a *P* value <  .05). Data were analyzed from December 2022 through January 2023.

## Results

Among 2 748 585 Korean adults (738 934 aged 50-64 years [29.1%] and 657 560 aged ≥65 years [25.9%]; 1 178 869 males [46.4%]) who participated in the KCHS from 2009 to 2021, there were 1 766 178 rural residents (69.4%), 1 787 178 individuals (70.3%) who had completed high school education or below, and 706 127 individuals (27.8%) who were classified at the top income level (within quartile 4). A total of 892 272 participants (35.1%) met physical activity guidelines recommended by WHO (ie, ≥600 MET-min/wk). Demographic characteristics of participants are presented in Table 1. There was no change in the prevalence of adults meeting the WHO-recommended minimum threshold for physical activity prior to the pandemic (β difference, 1.0; 95% CI, 0.6 to 1.4). After the onset of the COVID-19 pandemic, prevalence of physical activity decreased across all groups. Prevalence of physical activity decreased from 36.0% (95% CI, 35.9% to 36.1%) in 2017 to 2019 to 30.0% (95% CI, 29.8% to 30.2%) in 2020 and 29.7% (95% CI, 29.5% to 29.9%) in 2021 after the onset of the pandemic (β difference for trend after pandemic onset vs before the pandemic, −11.6; 95% CI, −12.1 to −10.3). As shown in the Figure and Table 2, this trend in physical activity was similar for middle-aged groups during the pandemic (ages 40 to 49 years: β difference, −8.4; 95% CI, −9.6 to −7.1; ages 50 to 64 years: β difference, −10.2; 95% CI, −11.1 to −9.2). Additionally, we conducted linear, quadratic, and cubic spline interpolations over all time periods to examine linear and nonlinear trends in physical activity (eFigure 1 in Supplement 1) and changes in prevalence using a heat map to visualize local shifts (eFigure 2 in Supplement 1).

**Table 1.** Demographic Characteristics

Characteristic	Participants, No. (%) (N = 2 543 652)
Age, y	
19-29	292 629 (11.5)
30-39	377 523 (14.8)
40-49	477 006 (18.8)
50-64	738 934 (29.1)
≥65	657 560 (25.9)
Sex	
Male	1 178 869 (46.4)
Female	1 364 783 (53.6)
BMI^a^	
Underweight	128 546 (5.1)
Normal	1 113 008 (43.8)
Overweight	622 333 (24.5)
Obese	679 765 (26.7)
Region of residence	
Urban	777 474 (30.6)
Rural	1 766 178 (69.4)
Educational level	
High school or below	1 787 178 (70.3)
College or above	756 474 (29.7)
Income level	
Quartile 1 (lowest)	545 952 (21.5)
Quartile 2	508 436 (20.0)
Quartile 3	783 137 (30.8)
Quartile 4 (highest)	706 127 (27.8)
Smoking status	
Current smoker	500 840 (19.7)
Former smoker	445 569 (17.5)
Never smoked	1 597 243 (62.8)
Alcohol intake, d/mo	
<1	1 241 776 (48.8)
1-4	763 899 (30.0)
≥5	537 977 (21.2)
Diabetes	
Yes	246 421 (9.7)
No	2 297 231 (90.3)
Hypertension	
Yes	618 833 (24.3)
No	1 924 819 (75.7)
Depression	
Yes	28 503 (1.1)
No	2 515 149 (98.9)
Stress status^b^	
Yes	47 902 (1.9)
No	2 495 750 (98.1)

Abbreviation: BMI, body mass index (calculated as weight in kilograms divided by height in meters squared).

^a^ According to Asia-Pacific guidelines, BMI is divided into 4 groups: underweight (<18.5), normal (18.5-22.9), overweight (23.0-24.9), and obese (≥25).

^b^ Stress was defined by receipt of mental health counseling owing to stress.

**Figure.**
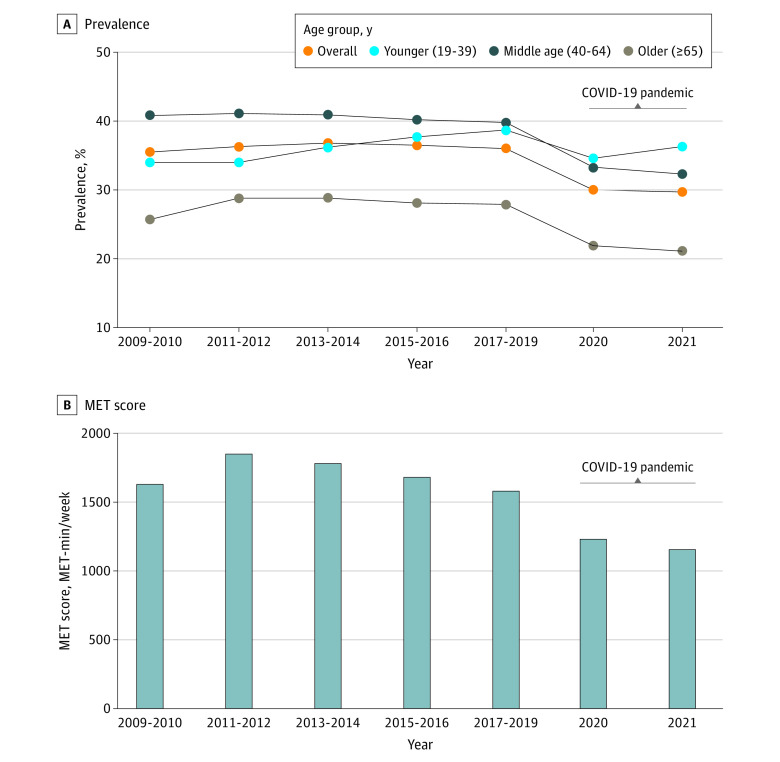
Prevalence and Metabolic Equivalent of Task (MET) Score of Physical Activity

**Table 2.** Prevalence and Trend of Sufficient Aerobic PA

Group	Prevalence, % (95% CI)								PA trend, β (95% CI)^a^		PA trend difference, β difference (95% CI)^a^	PA odds, OR (95% CI)^b^
	Before pandemic						After pandemic onset					
	2009-2010	2011-2012	2013-2014	2015-2016	2017-2019	2020		2021	Before pandemic	After pandemic onset		
Overall	35.5 (35.4 to 35.6)	36.3 (36.2 to 36.4)	36.8 (36.7 to 36.9)	36.5 (36.4 to 36.6)	36.0 (35.9 to 36.1)	30.0 (29.8 to 30.2)		29.7 (29.5 to 29.9)	1.0 (0.6 to 1.4)	−10.6 (−11.0 to −10.3)	−11.6 (−12.1 to −11.1)	0.76 (0.75 to 0.76)
Age, y												
19-29	33.9 (33.5 to 34.3)	35.1 (34.7 to 35.5)	38.1 (37.7 to 38.5)	39.2 (38.8 to 39.6)	40.1 (39.6 to 40.6)	36.6 (36.0 to 37.2)		39.1 (38.5 to 39.7)	14.3 (13.1 to 15.5)	−2.2 (−3.3 to −1.2)	−16.6 (−18.1 to −15.0)	0.91 (0.89 to 0.93)
30-39	34.1 (33.8 to 34.4)	33.1 (32.7 to 33.5)	34.8 (34.4 to 35.2)	36.6 (36.2 to 37.0)	37.5 (37.1 to 37.9)	32.6 (32.0 to 33.2)		33.7 (33.1 to 34.3)	8.5 (7.5 to 9.5)	−6.5 (−7.6 to −5.5)	−15.0 (−16.5 to −13.5)	0.83 (0.81 to 0.85)
40-49	41.4 (41.1 to 41.7)	40.4 (40.1 to 40.7)	40.5 (40.2 to 40.8)	40.5 (40.2 to 40.8)	39.9 (39.5 to 40.3)	33.6 (33.1 to 34.1)		33.3 (32.8 to 33.8)	−2.3 (−3.1 to −1.4)	−10.7 (−11.5 to −9.8)	−8.4 (−9.6 to −7.1)	0.76 (0.74 to 0.77)
50-64	40.3 (40.0 to 40.6)	41.6 (41.3 to 41.9)	41.2 (40.9 to 41.5)	40 (39.7 to 40.3)	39.7 (39.4 to 40.0)	33.2 (32.8 to 33.6)		31.8 (31.4 to 32.2)	−2.4 (−3.2 to −1.7)	−12.6 (−13.3 to −11.9)	−10.2 (−11.1 to −9.2)	0.73 (0.72 to 0.74)
≥65	25.7 (25.4 to 26.0)	28.8 (28.5 to 29.1)	28.8 (28.5 to 29.1)	28.1 (27.8 to 28.4)	27.9 (27.7 to 28.1)	21.9 (21.6 to 22.2)		21.1 (20.8 to 21.4)	3.1 (2.2 to 3.9)	−13.4 (−14.1 to −12.6)	−16.4 (−17.5 to −15.3)	0.71 (0.70 to 0.72)
Sex												
Male	43.4 (43.2 to 43.6)	43.6 (43.4 to 43.8)	43.7 (43.5 to 43.9)	43.1 (42.9 to 43.3)	42.6 (42.4 to 42.8)	37.6 (37.3 to 37.9)		36.5 (36.2 to 36.8)	−1.6 (−2.2 to −1.1)	−9.3 (−9.8 to −8.8)	−7.7 (−8.4 to −6.9)	0.79 (0.78 to 0.80)
Female	28.5 (28.3 to 28.7)	30.0 (29.8 to 30.2)	30.7 (30.5 to 30.9)	30.6 (30.4 to 30.8)	30.4 (30.2 to 30.6)	23.7 (23.5 to 23.9)		23.8 (23.6 to 24)	4.3 (3.7 to 4.8)	−12.5 (−13.1 to −12.0)	−16.8 (−17.6 to −16.0)	0.71 (0.70 to 0.72)
BMI^c^												
Underweight	22.3 (21.8 to 22.8)	24.4 (23.8 to 25.0)	25.5 (24.9 to 26.1)	25.2 (24.6 to 25.8)	25.3 (24.7 to 25.9)	20.8 (20.0 to 21.6)		20.8 (20.0 to 21.6)	7.5 (5.6 to 9.4)	−9.7 (−11.6 to −7.7)	−17.1 (−19.9 to −14.4)	0.78 (0.74 to 0.81)
Normal	34.1 (33.9 to 34.3)	35.0 (34.8 to 35.2)	35.5 (35.3 to 35.7)	35.2 (35.0 to 35.4)	34.8 (34.6 to 35.0)	28.6 (28.3 to 28.9)		28.4 (28.1 to 28.7)	1.5 (0.9 to 2.1)	−11.1 (−11.6 to −10.5)	−12.5 (−13.4 to −11.7)	0.75 (0.74 to 0.76)
Overweight	38.8 (38.5 to 39.1)	39.3 (39.0 to 39.6)	39.6 (39.3 to 39.9)	38.7 (38.4 to 39.0)	38.2 (37.9 to 38.5)	32.4 (32.0 to 32.8)		30.9 (30.5 to 31.3)	−1.6 (−2.4 to −0.8)	−11.7 (−12.4 to −10.9)	−10.0 (−11.1 to −9.0)	0.75 (0.74 to 0.76)
Obese	38.0 (37.7 to 38.3)	38.6 (38.3 to 38.9)	38.8 (38.5 to 39.1)	38.6 (38.3 to 38.9)	37.2 (36.9 to 37.5)	31.0 (30.7 to 31.3)		31.6 (31.2 to 32.0)	−1.6 (−2.4 to −0.8)	−9.5 (−10.2 to −8.9)	−7.9 (−8.9 to −6.9)	0.77 (0.75 to 0.78)
Region of residence,												
Urban	32.7 (32.4 to 33.0)	33.5 (33.2 to 33.8)	34.3 (34.0 to 34.6)	35.6 (35.3 to 35.9)	35.7 (35.4 to 36.0)	28.2 (27.9 to 28.5)		27.5 (27.2 to 27.8)	7.2 (6.4 to 7.9)	−14.1 (−14.7 to −13.4)	−21.2 (−22.2 to −20.2)	0.70 (0.68 to 0.71)
Rural	36.7 (36.5 to 36.9)	37.6 (37.4 to 37.8)	37.9 (37.7 to 38.1)	36.8 (36.6 to 37.0)	36.1 (35.9 to 36.3)	30.7 (30.5 to 30.9)		30.6 (30.4 to 30.8)	−1.7 (−2.2 to −1.2)	−9.2 (−9.6 to −8.8)	−7.5 (−8.1 to −6.8)	0.78 (0.78 to 0.79)
Educational level												
≤High school	34.6 (34.4 to 34.8)	35.9 (35.7 to 36.1)	35.7 (35.5 to 35.9)	34.8 (34.6 to 35.0)	33.7 (33.5 to 33.9)	27.6 (27.4 to 27.8)		26.7 (26.5 to 26.9)	−2.5 (−3.0 to −2.0)	−12.1 (−12.6 to −11.7)	−9.6 (−10.2 to −8.9)	0.73 (0.73 to 0.74)
≥College	37.8 (37.5 to 38.1)	37.5 (37.2 to 37.8)	39.2 (38.9 to 39.5)	40.2 (39.9 to 40.5)	40.9 (40.6 to 41.2)	35.1 (34.7 to 35.5)		35.5 (35.2 to 35.8)	7.5 (6.8 to 8.2)	−8.9 (−9.5 to −8.2)	−16.3 (−17.3 to −15.4)	0.79 (0.78 to 0.80)
Income level												
Quartile 1 (lowest)	27.8 (27.5 to 28.1)	28.8 (28.5 to 29.1)	28.3 (28.0 to 28.6)	27.2 (26.9 to 27.5)	26.3 (26.0 to 26.6)	21.4 (21.1 to 21.7)		20.4 (20.1 to 20.7)	−4.7 (−5.7 to −3.8)	−11.8 (−12.6 to −10.9)	−7.0 (−8.3 to −5.7)	0.74 (0.73 to 0.76)
Quartile 2	34.7 (34.4 to 35.0)	35.0 (34.7 to 35.3)	35.6 (35.3 to 35.9)	36.2 (35.8 to 36.6)	35.9 (35.6 to 36.2)	30.4 (30.0 to 30.8)		29.5 (29.1 to 29.9)	3.2 (2.3 to 4.1)	−10.5 (−11.3 to −9.7)	−13.7 (−14.9 to −12.4)	0.77 (0.75 to 0.78)
Quartile 3	37.1 (36.8 to 37.4)	37.3 (37 to 37.6)	38.1 (37.9 to 38.3)	38.1 (37.9 to 38.3)	38.2 (37.9 to 38.5)	32.8 (32.4 to 33.2)		32.1 (31.7 to 32.5)	2.3 (1.6 to 3.0)	−9.6 (−10.3 to −8.9)	−11.9 (−12.8 to −10.9)	0.79 (0.77 to 0.79)
Quartile 4 (highest)	41.1 (40.8 to 41.4)	42.3 (42 to 42.6)	42.2 (41.9 to 42.5)	40.8 (40.5 to 41.1)	40.7 (40.4 to 41)	34.9 (34.5 to 35.3)		34.9 (34.5 to 35.3)	−2.1 (−2.8 to −1.3)	−9.3 (−10.0 to −8.7)	−7.2 (−8.2 to −6.2)	0.78 (0.77 to 0.79)
Smoking status												
Current smoker	41.0 (40.7 to 41.3)	41.5 (41.2 to 41.8)	41.7 (41.4 to 42.0)	41.0 (40.7 to 41.3)	40.2 (39.8 to 40.6)	36.0 (35.5 to 36.5)		34.5 (34.0 to 35.0)	−1.4 (−2.3 to −0.6)	−8.8 (−9.6 to −7.9)	−7.3 (−8.5 to −6.1)	0.81 (0.79 to 0.83)
Former smoker	42.3 (41.9 to 42.7)	42.5 (42.1 to 42.9)	42.4 (42.0 to 42.8)	41.6 (41.3 to 41.9)	41.1 (40.8 to 41.4)	36.0 (35.5 to 36.5)		34.9 (34.4 to 35.4)	−2.8 (−3.7 to −1.8)	−9.7 (−10.5 to −8.9)	−6.9 (−8.2 to −5.7)	0.79 (0.77 to 0.80)
Never smoked	31.8 (31.6 to 32.0)	32.9 (32.7 to 33.1)	33.6 (33.4 to 33.8)	33.5 (33.3 to 33.7)	33.2 (33.0 to 33.4)	26.8 (26.6 to 27.0)		26.9 (26.7 to 27.1)	3.0 (2.5 to 3.6)	−11.3 (−11.7 to −10.8)	−14.3 (−15.0 to −13.6)	0.74 (0.73 to 0.75)
Alcohol intake, d/mo												
<1	29.8 (29.6 to 30.0)	31.0 (30.8 to 31.2)	31.3 (31.1 to 31.5)	30.7 (30.5 to 30.9)	30.6 (30.4 to 30.8)	25.2 (25.0 to 25.4)		24.8 (24.6 to 25)	1.1 (0.5 to 1.7)	−10.8 (−11.3 to −10.2)	−11.9 (−12.7 to −11.1)	0.76 (0.75 to 0.77)
1-4	38.5 (38.2 to 38.8)	38.7 (38.4 to 39.0)	39.7 (39.4 to 40.0)	39.8 (39.5 to 40.1)	40.0 (39.7 to 40.3)	35.0 (34.6 to 35.4)		35.5 (35.1 to 35.9)	3.4 (2.7 to 4.1)	−7.2 (−7.8 to −6.5)	−10.6 (−11.6 to −9.7)	0.82 (0.81 to 0.83)
≥5	44.2 (43.9 to 44.5)	44.5 (44.2 to 44.8)	44.0 (43.7 to 44.3)	43.5 (43.2 to 43.8)	42.6 (42.3 to 42.9)	37.2 (36.7 to 37.7)		36.4 (35.9 to 36.9)	−3.4 (−4.2 to −2.6)	−9.4 (−10.2 to −8.6)	−6.0 (−7.1 to −4.9)	0.79 (0.77 to 0.80)
Diabetes												
Yes	29.8 (29.3 to 30.3)	31.7 (31.2 to 32.2)	31.4 (30.9 to 31.9)	30.2 (29.8 to 30.6)	30.1 (29.7 to 30.5)	24.1 (23.6 to 24.6)		23.4 (22.9 to 23.9)	−1.3 (−2.7 to 0.1)	−12.7 (−13.8 to −11.5)	−11.4 (−13.2 to −9.6)	0.72 (0.70 to 0.74)
No	35.9 (35.7 to 36.1)	36.7 (36.5 to 36.9)	37.3 (37.1 to 37.5)	37.1 (36.9 to 37.3)	36.7 (36.5 to 36.9)	30.8 (30.6 to 31.0)		30.5 (30.3 to 30.7)	1.7 (1.3 to 2.1)	−10.3 (−10.7 to −9.9)	−12.0 (−12.6 to −11.4)	0.76 (0.76 to 0.77)
Hypertension												
Yes	31.0 (30.7 to 31.3)	32.9 (32.6 to 33.2)	33.2 (32.9 to 33.5)	32.2 (31.9 to 32.5)	31.1 (30.8 to 31.4)	25.2 (24.9 to 25.5)		24.5 (24.2 to 24.8)	−1.1 (−1.9 to −0.2)	−12.1 (−12.8 to −11.4)	−11.0 (−12.1 to −9.9)	0.73 (0.72 to 0.75)
No	36.6 (36.4 to 36.8)	37.3 (37.1 to 37.5)	37.8 (37.6 to 38)	37.9 (37.7 to 38.1)	37.8 (37.6 to 38)	31.9 (31.7 to 32.1)		31.7 (31.5 to 31.9)	2.6 (2.2 to 3.1)	−10.1 (−10.5 to −9.6)	−12.7 (−13.3 to −12.1)	0.77 (0.76 to 0.77)
Depression												
Yes	29.6 (28.2 to 31)	30.8 (29.3 to 32.3)	30.9 (29.5 to 32.3)	32.1 (30.8 to 33.4)	30.8 (29.5 to 32.1)	26.3 (24.7 to 27.9)		25.2 (23.8 to 26.6)	3.2 (−0.8 to 7.3)	−10.5 (−13.9 to −7.1)	−13.7 (−19.1 to −8.4)	0.78 (0.72 to 0.84)
No	35.5 (35.4 to 35.6)	36.4 (36.3 to 36.5)	36.8 (36.7 to 36.9)	36.5 (36.4 to 36.6)	36 (35.9 to 36.1)	30.0 (29.8 to 30.2)		29.7 (29.5 to 29.9)	1.0 (0.6 to 1.4)	−10.6 (−11.0 to −10.2)	−11.6 (−12.1 to −11.0)	0.76 (0.75 to 0.76)
Stress status^d^												
Yes	31.3 (30.1 to 32.5)	33.3 (32.1 to 34.5)	32.8 (31.7 to 33.9)	34.1 (33.1 to 35.1)	33.6 (32.6 to 34.6)	29.1 (27.9 to 30.3)		29.1 (27.9 to 30.3)	4.5 (1.5 to 7.5)	−7.9 (−10.4 to −5.3)	−12.4 (−16.3 to −8.4)	0.81 (0.76 to 0.86)
No	35.5 (35.4 to 35.6)	36.4 (36.3 to 36.5)	36.8 (36.7 to 36.9)	36.5 (36.4 to 36.6)	36.0 (35.9 to 36.1)	30.0 (29.8 to 30.2)		29.7 (29.5 to 29.9)	0.9 (05 to 1.3)	−10.7 (−11.1 to −10.3)	−11.6 (−12.2 to −11.1)	0.76 (0.75 to 0.76)

Abbreviations: BMI, body mass index (calculated as weight in kilograms divided by height in meters squared); OR, odds ratio; PA, physical activity.

^a^ The β values were multiplied by 100 owing to their small values; they were considered significant when their 95% CIs did not cross 0.

^b^ The model was adjusted for age, sex, region of residence, BMI, educational level, income level, smoking status, alcohol intake, and diabetes, hypertension, depression, and stress status. ORs compare the pandemic period (2020-2021) with the prepandemic period (2017-2019, the reference value). ORs were considered significant when their 95% CIs did not cross 1.

^c^ According to Asia-Pacific guidelines, BMI is divided into 4 groups: underweight (<18.5), normal (18.5-22.9), overweight (23.0-24.9), and obese (≥25).

^d^ Stress was defined by receipt of mental health counseling owing to stress.

Physical activity trends showed significant decreases in older adults (ages ≥65 years; β difference, −16.4; 95% CI, −17.5 to −15.3) and younger adults (ages 19 to 29: β difference, −16.6; 95% CI, −18.1 to −15.0; ages 30 to 39 years: β difference, −15.0; 95% CI, −16.5 to −13.5) during the pandemic. In younger adults, however, the prevalence of physical activity increased during the midpandemic vs early pandemic period for those ages 19 to 29 years (39.1%; 95% CI, 38.5% to 39.7% vs 36.6%; 95% CI, 36.0% to 37.2%) and ages 30 to 39 years (33.7%; 95% CI, 33.1% to 34.3% vs 32.6%; 95% CI, 32.0% to 33.2%).

The prevalence of physical activity declined from before to during the pandemic, particularly among females (β difference, −16.8; 95% CI, −17.6 to −16.0), participants who lived in urban residences (β difference, −21.2; 95% CI, −22.2 to −20.2), and middle class groups(50% of the middle class [income level quartile 3]: β difference, −11.9; 95% CI, −12.8 to −10.9; 75% of the middle class [income level quartile 2]: β difference, −13.7; 95% CI, −14.9 to −12.4). Healthy participants, including those with low BMI (eg, normal BMI: β difference,−12.5; 95% CI, −13.4 to −11.7), as well as nonsmokers, nondrinkers, and those with no history of chronic medical conditions, such as diabetes and hypertension, also had large decreases, as did individuals at increased risk of stress, such as those with history of a depressive episode (β difference, −13.7; 95% CI, −19.1 to −8.4) and high stress status.

The total MET score of physical activity showed a similar tendency to that of physical activity (Table 3). The overall mean MET score decreased from the 2017 to 2019 period (1579.1 MET-min/wk; 95% CI, 1567.5 to 1590.7 MET-min/wk) to the 2020 to 2021 period (1191.9 MET-min/wk; 95% CI, 1182.4 to 1201.4 MET-min/wk). The mean difference in MET between the 2 periods was −387.2 MET-min/wk (95% CI, −402.2 to −372.1 MET-min/wk). Concerning the total MET score with a β-difference, most defined groups showed a decreasing trend between prepandemic and early and midpandemic periods. However, there was a difference between high-income groups (quartile 4 [highest]) during the COVID-19 pandemic. The total mean MET score trends with sufficient physical activity from 2020 to 2021 increased in the high economic-level group compared with the prepandemic period (β difference, 3.7; 95% CI, 3.4 to 4.0); this was not observed in other groups.

**Table 3.** Mean MET Score for Moderate- and Vigorous-Intensity PA

Group	MET score, mean (95% CI), MET-min/wk								PA trend, β (95% CI)^a^		PA trend difference, β difference (95% CI)^a^
	Before pandemic					After pandemic onset			Before pandemic	After pandemic onset	
	2009-2010	2011-2012	2013-2014	2015-2016	2017-2019		2020	2021			
Overall	1629.4 (1617.4 to 1641.4)	1849.4 (1835.7 to 1863.0)	1780.7 (1767.7 to 1793.6)	1678.9 (1666.6 to 1691.2)	1579.1 (1567.5 to 1590.7)		1228.7 (1215.0 to 1242.5)	1154.9 (1141.8 to 1168.1)	−0.7 (−0.8 to −0.6)	−2.0 (−2.1 to −2.0)	−1.3 (−1.4 to −1.2)
Age, y											
19-29	1169.2 (1144.9 to 1193.5)	1275.7 (1247.5 to 1304.0)	1348.3 (1320.2 to 1376.3)	1366.2 (1338.9 to 1393.6)	1357.1 (1329.4 to 1384.7)		1179.9 (1147.2 to 1212.5)	1259.6 (1223.9 to 1295.3)	2.2 (1.8 to 2.6)	−1.0 (−1.3 to −0.7)	−3.2 (−3.7 to −2.7)
30-39	1307.4 (1283.8 to 1331.1)	1343.4 (1317.3 to 1369.5)	1313.3 (1287.9 to 1338.7)	1407.9 (1380.1 to 1435.8)	1366.3 (1338.0 to 1394.6)		1163.0 (1124.1 to 1201.9)	1116.4 (1080.7 to 1152.1)	0.7 (0.4 to 1.0)	−1.3 (−1.6 to −1.0)	−2.0 (−2.4 to −1.6)
40-49	1840.4 (1812.9 to 1867.9)	1897.6 (1868.2 to 1927.0)	1771.6 (1743.6 to 1799.6)	1699.2 (1671.2 to 1727.3)	1603.1 (1575.3 to 1630.9)		1318.8 (1282.5 to 1355.0)	1183.0 (1150.1 to 1216.0)	−1.6 (−1.8 to −1.4)	−1.8 (−2.1 to −1.6)	−0.2 (−0.5 to 0.1)
50-64	2131.9 (2104.2 to 2159.6)	2473.8 (2443.1 to 2504.5)	2334.2 (2305.4 to 2363.1)	2111.9 (2085.6 to 2138.2)	1976.8 (1952.0 to 2001.6)		1515.3 (1486.3 to 1544.3)	1371.4 (1344.1 to 1398.6)	−1.3 (−1.5 to −1.2)	−2.2 (−2.3 to −2.0)	−0.9 (−1.1 to −0.7)
≥65	1358.0 (1332.9 to 1383.0)	1689.3 (1660.6 to 1718.0)	1631.3 (1604.9 to 1657.8)	1451.3 (1428.2 to 1474.5)	1333.0 (1313.1 to 1352.8)		954.4 (932.7 to 976.2)	911.6 (890.2 to 932.9)	−1.1 (−1.3 to −0.9)	−2.4 (−2.6 to −2.2)	−1.3 (−1.6 to −1.0)
Sex											
Male	2066.7 (2047.0 to 2086.3)	2289.0 (2266.9 to 2311.1)	2186.0 (2165.1 to 2206.9)	2090.1 (2070.0 to 2110.3)	1990.6 (1971.3 to 2009.9)		1658.7 (1634.6 to 1682.8)	1513.2 (1490.8 to 1535.6)	−0.7 (−0.9 to −0.6)	−1.7 (−1.8 to −1.6)	−1.0 (−1.2 to −0.8)
Female	1241.9 (1227.5 to 1256.3)	1466.7 (1450.0 to 1483.3)	1425.2 (1409.3 to 1441.1)	1321.5 (1306.8 to 1336.2)	1233.0 (1219.2 to 1246.7)		872.4 (857.3 to 887.4)	848.5 (833.6 to 863.5)	−0.6 (−0.7 to −0.4)	−2.6 (−2.8 to −2.5)	−2.0 (−2.2 to −1.8)
BMI^b^											
Underweight	1037.3 (995.8 to 1078.7)	1247.8 (1198.9 to 1296.8)	1231.2 (1183.1 to 1279.3)	1135.0 (1090.0 to 1180.0)	1072.0 (1025.7 to 1118.2)		794.4 (739.6 to 849.3)	732.8 (682.2 to 783.5)	−0.1 (−0.6 to 0.4)	−2.4 (−2.9 to −1.9)	−2.3 (−3.0 to −1.6)
Normal	1547.5 (1530.4 to 1564.7)	1777.6 (1757.9 to 1797.4)	1695.9 (1677.1 to 1714.7)	1587.2 (1569.1 to 1605.3)	1481.0 (1463.4 to 1498.7)		1126.4 (1105.8 to 1147.0)	1071.6 (1051.9 to 1091.2)	−0.8 (−0.9 to −0.6)	−2.3 (−2.4 to −2.1)	−1.5 (−1.7 to −1.3)
Overweight	1812.7 (1786.9 to 1838.4)	2007.0 (1978.5 to 2035.5)	1927.0 (1899.9 to 1954.2)	1809.6 (1784.1 to 1835.2)	1700.0 (1675.7 to 1724.4)		1359.9 (1330.1 to 1389.6)	1222.4 (1195.2 to 1249.6)	−1.0 (−1.2 to −0.8)	−2.1 (−2.3 to −1.9)	−1.1 (−1.4 to −0.8)
Obese	1751.9 (1726.0 to 1777.7)	1968.2 (1939.4 to 1997.0)	1912.3 (1885.4 to 1939.3)	1811.7 (1787.0 to 1836.4)	1683.3 (1661.7 to 1705.0)		1312.3 (1287.0 to 1337.6)	1273.7 (1248.3 to 1299.1)	−0.9 (−1.1 to −0.7)	−1.8 (−2.0 to −1.7)	−0.9 (−1.2 to −0.7)
Region of residence											
Urban	1119.4 (1103.6 to 1135.1)	1221.4 (1203.9 to 1239.0)	1218.9 (1202.1 to 1235.7)	1254.0 (1237.1 to 1270.8)	1189.2 (1173.7 to 1204.8)		892.0 (873.4 to 910.7)	823.4 (806.2 to 840.6)	0.6 (0.4 to 0.9)	−3.1 (−3.4 to −2.9)	−3.7 (−4.1 to −3.3)
Rural	1852.3 (1836.5 to 1868.1)	2132.7 (2114.6 to 2150.7)	2034.0 (2016.9 to 2051.2)	1869.1 (1853.0 to 1885.1)	1747.2 (1732.0 to 1762.3)		1369.6 (1351.7 to 1387.4)	1298.2 (1280.9 to 1315.4)	−1.0 (−1.1 to −0.9)	−1.9 (−1.9 to −1.8)	−0.9 (−1.0 to −0.8)
Educational level											
≤High school	1789.3 (1774.0 to 1804.6)	2077.7 (2060.0 to 2095.3)	1992.4 (1975.3 to 2009.4)	1843.5 (1827.2 to 1859.7)	1698.2 (1682.9 to 1713.5)		1287.5 (1269.6 to 1305.5)	1200.8 (1183.2 to 1218.3)	−0.9 (−1.0 to −0.8)	−2.1 (−2.2 to −2.0)	−1.2 (−1.3 to −1.1)
≥College	1178.1 (1162.3 to 1194.0)	1258.4 (1241.2 to 1275.6)	1275.4 (1259.1 to 1291.6)	1311.5 (1295.5 to 1327.4)	1314.1 (1298.5 to 1329.8)		1099.8 (1080.2 to 1119.4)	1065.2 (1047.1 to 1083.3)	1.6 (1.3 to 1.9)	−1.8 (−2.0 to −1.6)	−3.4 (−3.8 to −3.0)
Income level											
Quartile 1 (lowest)	1454.1 (1429.1 to 1479.1)	1602.1 (1574.1 to 1630.2)	1623.8 (1594.7 to 1652.9)	1513.8 (1485.2 to 1542.3)	1323.5 (1298.9 to 1348.0)		940.2 (915.4 to 965.1)	889.0 (863.4 to 914.5)	−0.9 (−1.1 to −0.7)	−2.1 (−2.3 to −1.9)	−1.2 (−1.5 to −0.9)
Quartile 2	1575.7 (1551.3 to 1600.1)	1779.3 (1749.9 to 1808.8)	1942.6 (1909.4 to 1975.9)	2050.7 (2015.6 to 2085.9)	1852.9 (1822.3 to 1883.4)		1406.2 (1373.4 to 1438.9)	1334.8 (1303.3 to 1366.4)	1.9 (1.7 to 2.1)	−2.0 (−2.1 to −1.8)	−3.9 (−4.2 to −3.7)
Quartile 3	1410.6 (1391.7 to 1429.5)	1524.1 (1504.1 to 1544.2)	1631.2 (1611.5 to 1650.9)	1755.3 (1734.2 to 1776.5)	1712.4 (1690.1 to 1734.8)		1365.9 (1336.8 to 1395.1)	1245.2 (1217.8 to 1272.6)	2.2 (2.0 to 2.4)	−2.1 (−2.3 to −2.0)	−4.3 (−4.6 to −4.1)
Quartile 4 (highest)	2079.8 (2051.8 to 2107.9)	2491.5 (2459.3 to 2523.7)	1987.3 (1960.8 to 2013.8)	1477.1 (1458.1 to 1496.1)	1454.3 (1436.3 to 1472.4)		1220.7 (1196.8 to 1244.5)	1154.4 (1132.6 to 1176.2)	−5.4 (−5.6 to −5.2)	−1.7 (−1.9 to −1.5)	3.7 (3.4 to 4.0)
Smoking status											
Current smoker	2097.5 (2068.3 to 2126.7)	2370.5 (2336.2 to 2404.8)	2302.0 (2268.4 to 2335.6)	2243.1 (2208.2 to 2277.9)	2150.1 (2115.3 to 2184.9)		1840.3 (1794.7 to 1885.8)	1676.4 (1632.9 to 1720.0)	0.1 (−0.1 to 0.2)	−1.3 (−1.4 to −1.1)	−1.4 (−1.6 to −1.2)
Former smoker	1933.8 (1900.2 to 1967.3)	2134.9 (2099.8 to 2170.0)	2044.4 (2011.8 to 2077.1)	1925.8 (1896.4 to 1955.1)	1828.1 (1800.6 to 1855.7)		1488.6 (1454.2 to 1522.9)	1350.0 (1318.9 to 1381.1)	−1.2 (−1.4 to −0.9)	−2.0 (−2.2 to −1.9)	−0.8 (−1.1 to −0.5)
Never smoked	1384.0 (1370.1 to 1397.8)	1590.3 (1574.4 to 1606.2)	1533.2 (1518.1 to 1548.2)	1433.2 (1419.2 to 1447.2)	1344.7 (1331.5 to 1357.8)		1003.2 (988.5 to 1018.0)	965.7 (951.3 to 980.0)	−0.8 (−0.9 to −0.6)	−2.4 (−2.5 to −23)	−1.6 (−1.8 to −1.4)
Alcohol intake, d/mo											
<1	1425.4 (1408.9 to 1442.0)	1679.4 (1659.9 to 1698.9)	1612.3 (1593.6 to 1631.0)	1475.4 (1458.1 to 1492.7)	1389.0 (1373.0 to 1405.0)		1043.2 (1026.0 to 1060.4)	990.5 (974.0 to 1007.0)	−0.8 (−0.9 to 0.0)	−2.1 (−2.3 to −2.0)	−1.3 (−1.8 to −0.8)
1-4	1534.3 (1514.7 to 1553.9)	1669.3 (1647.5 to 1691.0)	1626.7 (1606.1 to 1647.3)	1572.8 (1553.2 to 1592.3)	1514.6 (1495.5 to 1533.7)		1270.4 (1244.9 to 1295.9)	1201.3 (1177.5 to 1225.1)	−0.4 (−0.6 to −0.2)	−1.7 (−1.8 to −1.5)	−1.3 (−1.6 to −1.1)
≥5	2254.5 (2222.7 to 2286.4)	2468.7 (2434.7 to 2502.8)	2340.9 (2309.0 to 2372.7)	2242.8 (2212.0 to 2273.6)	2093.4 (2063.8 to 2123.1)		1737.3 (1697.4 to 1777.2)	1604.7 (1565.9 to 1643.5)	−1.1 (−1.2 to −0.9)	−1.6 (−1.7 to −1.4)	−0.5 (−0.7 to −0.3)
Diabetes											
Yes	1454.2 (1411.1 to 1497.2)	1711.2 (1665.0 to 1757.3)	1637.5 (1595.3 to 1679.6)	1521.6 (1483.7 to 1559.5)	1443.7 (1408.8 to 1478.5)		1053.7 (1015.5 to 1092.0)	1010.1 (973.4 to 1046.8)	−0.8 (−1.1 to −0.4)	−2.2 (−2.4 to −1.9)	−1.4 (−1.8 to −1.0)
No	1643.4 (1630.9 to 1656.0)	1861.8 (1847.5 to 1876.2)	1794.9 (1781.2 to 1808.5)	1696.3 (1683.3 to 1709.3)	1596.1 (1583.8 to 1608.4)		1251.9 (1237.2 to 1266.7)	1175.3 (1161.2 to 1189.3)	−0.7 (−0.8 to −0.6)	−2.0 (−2.1 to −1.9)	−1.3 (−1.4 to −1.2)
Hypertension											
Yes	1519.3 (1492.5 to 1546.1)	1768.2 (1739.4 to 1797.0)	1734.2 (1707.1 to 1761.3)	1623.0 (1598.1 to 1647.9)	1481.7 (1459.5 to 1504.0)		1101.0 (1075.5 to 1126.6)	1044.5 (1020.1 to 1068.9)	−0.8 (−1.0 to −0.6)	−2.2 (−2.4 to −2.1)	−1.4 (−1.7 to −1.2)
No	1656.8 (1643.3 to 1670.3)	1872.0 (1856.5 to 1887.5)	1794.7 (1779.9 to 1809.5)	1697.3 (1683.2 to 1711.5)	1616.0 (1602.4 to 1629.6)		1278.3 (1261.9 to 1294.6)	1199.0 (1183.4 to 1214.6)	−0.6 (−0.7 to −0.5)	−2.0 (−2.1 to −1.9)	−1.4 (−1.5 to −1.3)
Depression											
Yes	1357.9 (1235.0 to 1480.8)	1519.4 (1385.9 to 1652.9)	1492.9 (1369.2 to 1616.5)	1518.8 (1405.4 to 1632.1)	1349.4 (1246.0 to 1452.7)		1092.7 (974.1 to 1211.3)	1003.7 (895.2 to 1112.2)	−0.1 (−1.2 to 0.9)	−1.7 (−2.5 to −1.0)	−1.6 (−2.9 to −0.3)
No	1632.0 (1619.9 to 1644.1)	1852.3 (1838.6 to 1866.1)	1783.7 (1770.6 to 1796.7)	1680.7 (1668.4 to 1693.1)	1581.9 (1570.2 to 1593.6)		1230.6 (1216.7 to 1244.4)	1157.5 (1144.2 to 1170.7)	−0.7 (−0.8 to −0.6)	−2.0 (−2.1 to −1.9)	−1.3 (−1.4 to −1.2)
Stress status^c^											
Yes	1468.2 (1367.6 to 1568.9)	1794.6 (1681.6 to 1907.7)	1629.5 (1533.5 to 1725.5)	1666.7 (1576.7 to 1756.7)	1521.9 (1436.2 to 1607.7)		1205.5 (1112.2 to 1298.7)	1146.9 (1058.5 to 1235.4)	−0.2 (−0.9 to 0.6)	−1.9 (−2.4 to −1.3)	−1.7 (−2.6 to −0.8)
No	1631.9 (1619.7 to 1644.0)	1850.3 (1836.5 to 1864.0)	1783.4 (1770.3 to 1796.5)	1679.2 (1666.8 to 1691.6)	1580.3 (1568.5 to 1592.0)		1229.3 (1215.3 to 1243.2)	1155.2 (1141.9 to 1168.5)	−0.7 (−0.8 to −0.6)	−2.0 (−2.1 to −2.0)	−1.3 (−1.4 to −1.2)

Abbreviations: BMI, body mass index; MET, metabolic equivalent of task; PA, physical activity.

^a^ The β values were multiplied by 100 000 owing to their minimal number; they were considered statistically significant when they did not cross 0.

^b^ According to Asia-Pacific guidelines, BMI is divided into 4 groups: underweight (<18.5), normal (18.5-22.9), overweight (23.0-24.9), and obese (≥25).

^c^ Stress was defined by receipt of mental health counseling owing to stress.

## Discussion

### Findings of This Study

This cross-sectional study found that before the pandemic, the prevalence of physical activity shown by each variable was stable or consistent. However, with the advent of the COVID-19 pandemic, the estimated prevalence rate of physical activity decreased, particularly among specific groups, such as older adults, females, participants living in urban residences, healthy individuals (normal BMI, nonsmoking, not drinking, and no history of chronic medical conditions such as diabetes or hypertension), and those at increased risk of stress (with depressive episodes and high-stress status). Interestingly, it was observed that only the younger group exhibited an increase in the prevalence of physical activity in 2021, during the middle of the pandemic, returning to prepandemic levels from a substantial decline after the onset of the COVID-19 pandemic.

### Comparison With Previous Studies

Several studies have been conducted on prevalence and trends of physical activity worldwide. Recently, studies have explored the association of the COVID-19 pandemic with adult physical activity levels. Studies on physical activity trends before COVID-19 showed an increase in Poland (7347 participants)^16^ and Australia (107 171 participants)^17^ and a decrease in the US (27 343 participants),^18^ Portugal (9856 participants),^19^ and Japan (286 704 participants).^20^ There were also stable research results in a population-based study (1.9 million participants) from 168 countries.^21^ During the pandemic, physical activity studies presented mixed results, with an increase in some countries, such as Spain (213 participants)^22^ and the United Kingdom (117 participants),^23^ and a decrease in others, such as Saudi Arabia (297 participants),^24^ Italy (400 participants),^25^ Canada (2338 participants),^26^ and South Korea (834 participants).^27^ Studies for some countries reported mixed findings, including for Switzerland (110 participants),^28^ Australia (60 560 participants),^29^ China (815 participants),^30^ and Japan (165 participants).^31^ However, a 2022 meta-analysis study^8^ found that physical activity decreased in all age groups, regardless of sex, although the change was not statistically significant in all age groups. Small sample sizes, diversity of populations across different nations, short-term follow-up times, and unsuitable study designs may have contributed to the weak level of evidence and inconsistent findings among studies in the meta-analysis (eTable in Supplement 1). Most studies were conducted in the US or Europe and reported changes in physical activity only until the early pandemic period in 2020. By contrast, our study provides physical activity and β coefficients to compare changes in physical activity levels in each period from 2009 to 2021.

### Possible Explanations of Study Results

Using a large-scale national-population data set, our analysis focused on tracking changes in prevalence and amount of physical activity. Various cultural, environmental, and social factors have been found to be associated with physical activity, which should be continuously assessed.^32^ Although people’s physical activity levels have decreased as technology has made life easier and more accessible, we found that the prevalence of physical activity remained stable and consistent from 2009 to 2019. This may be associated with WHO’s efforts to increase the level of physical activity globally and minimize the prevalence of physical inactivity, one of the goals of the WHO Global Action Plan from 2013 to 2020.^33^ We found that throughout the pandemic, the prevalence of physical activity decreased significantly and total MET scores also decreased. Confinement policies enforced by governments may have been associated with reduced daily activities (walking, cycling, and commuting to work) and increased time spent indoors at home.^34^ Forced, sudden inaccessibility to community resources, such as sports facilities, urban trails, and parks, was associated with the decline in vigorous activity.^35^

Physical activity trends in our study showed prominent decreases among older adults from before to during the pandemic period. In addition, the total MET score decreased significantly in older adults compared with younger groups. This is similar to findings from most previous studies,^36,37,38^ which reported decreased physical activity during the pandemic. Older adults faced an increased risk of developing severe COVID-19 and may have paid special attention to comply with government regulations and protect themselves in the best way.^8^

As in earlier investigations, we found that levels of physical activity differed between males and females; males were more active than females, and there was no significant change in physical activity compared with the period before the pandemic among males.^39^ The COVID-19 pandemic was associated with more changes in existing work experiences among females (such as reduced working hours and increased remote work and unemployment) compared with males, and females reported experiencing much more generalized anxiety.^40^ Anxiety and depressive symptoms may be associated with physical activity and mental health among females, suggesting that support for females may be needed after the onset of the COVID-19 pandemic.

Physical inactivity was more common at low than high income levels during the entire study period. This may have been associated with increased leisure time to exercise, which is more common in high-income than low-income groups.^41^ After the onset of the pandemic, the prevalence of physical activity decreased at all income levels; however, the difference in the physical activity tendency in the middle class was the largest and most prominent compared with the prepandemic period. This may have been associated with the decrease in occupational physical activity and leisure time exercise.^42^ Interestingly, at high income levels, the tendency of physical activity decreased less than expected compared with the prepandemic period. Individuals at high income levels may have had high levels of exercise through organized sports (eg, sports clubs) and may have received enhanced exercise support through virtual and online mediums during the pandemic.^43,44^

### Policy Implications

This study not only examined the overall prevalence of physical activity during the COVID-19 pandemic, but also considered the prevalence of physical activity and changes in physical activity levels among groups at high risk socially through subgroup analyses. This approach allowed us to better understand the association of the pandemic with activity in different segments of the population, including older adults, females, people living in urban residences, and low-income individuals. This analysis may also provide insights for policymakers and practitioners to effectively address challenges faced by these high-risk groups in maintaining physical activity levels during times of crisis.

During the COVID-19 pandemic, physical activity decreased across age groups, sex, and types of residence. Decreased physical activity during the pandemic could be associated with negative mental health and chronic disease outcomes.^40^ These findings suggest that socioeconomic and physiological factors may play a significant role in individual health behaviors.^45^ In response, governments may need to prioritize promoting physical activity and establishing a supportive environment during the pandemic. This may include approving and allowing physical activity and exercise within the guidelines of relevant safety and hygiene laws implemented due to the pandemic. It is important to emphasize that contactless outdoor sports activities were not found to be associated with any aerosol particle infection.^46^ Higher levels of physical activity may be associated with not only benefits in the general public's health, but also decreased medical treatment costs.

### Limitations and Strengths

This study has several limitations. First, self-reported information was used to evaluate physical activity, and recall bias may have occurred. Second, we did not use quantification measurement equipment, which made it challenging to accurately quantify the level of physical activity. Prior research^47^ found that users of digital platforms were more likely to follow recommendations for physical activity during the pandemic than nonusers. This suggests that bringing sports content to digital platforms during a pandemic should be considered. Further research using effective physical activity evaluation tools is required. Third, this study did not include data on physical activity from 2018. Consequently, there may be errors in the values of the trends in physical activity. Fourth, because this study included only South Korean adults, the findings do not accurately reflect the global population. Fifth, while this study included data from the prepandemic period and early to middle stages of the COVID-19 pandemic, ongoing monitoring is necessary to account for possible changes in physical activity trends in the later stages of the pandemic.

Despite these limitations, this study possesses several notable strengths. First, it used large-scale population-based data from a nationwide study that investigated physical activity trends among adults in South Korea. Second, long-term cross-sectional data from 2009 to 2021 were used, allowing for a comprehensive assessment of changes in physical activity over time. Third, in addition to examining differences between prepandemic and pandemic periods, we explored trends in physical activity during early and midpandemic phases. Fourth, this study identified trends in physical activity by various factors, such as region of residence, education level, income status, smoking status, alcohol consumption level, and diabetes, hypertension, and stress status, in addition to age and sex. These were important factors associated with physical activity during the pandemic period. Fifth, to our knowledge, this study was pioneering in its use of β difference and total MET score to quantify changes in physical activity from before to during the COVID-19 pandemic.

## Conclusions

To our knowledge, this study was the first long-term cross-sectional study and large-scale investigation of physical activity before and during the pandemic to use a representative population-based data set. It revealed trends in physical activity among more than 2 million South Korean individuals. This study found that the prevalence of partaking in physical activity was stable or consistent before the pandemic period but declined during the pandemic in 2020 and 2021. In particular, the trend of physical activity declined significantly among older adults, females, participants living in urban residences, healthy participants (ie, normal BMI, nonsmoking status, not drinking, and no history of chronic medical conditions, including diabetes and hypertension), and participants who were at increased risk of stress (history of depressive episodes and high-stress status) during the pandemic compared with the prepandemic period. Future studies should further evaluate the association between the COVID-19 pandemic and changes in physical activity.

## Notes

Supplement 1. **eFigure 1.** Linear and Nonlinear Trends of Physical Activity for All Time Periods **eFigure 2.** Rate of Change in Prevalence of Physical Activity Before and After COVID-19 Pandemic Onset **eTable.** Results of a Systematic Rapid Review Reporting Trends of Physical Activity in Study Periods

Supplement 2.
Data Sharing Statement
