# Supplementary material for: National Trends in Physical Activity Among Adults in South Korea Before and During the COVID-19 Pandemic, 2009-2021
Source: JAMA Netw Open. 2023 Jun 5;6(6):e2316930. doi: 10.1001/jamanetworkopen.2023.16930 (PMC10242425; doi:10.1001/jamanetworkopen.2023.16930)
Supplement: Supplement 2. — Data Sharing Statement [file jamanetwopen-e2316930-s002.pdf]

Data Sharing Statement

Park. National Trends in Physical Activity Among Adults in South Korea Before and During the COVID-19 Pandemic, 2009-2021. *JAMA Netw Open*. Published online June 5, 2023. doi:10.1001/jamanetworkopen.2023.16930

Data

Data available: Data are available on reasonable request. Study protocol, statistical code: available from DKY (email: yonkkang@gmail.com). Data set: available from the Korea Disease Control Agency (KDCA) through a data use agreement.

© 2023 Sangil Park et al. *JAMA Network Open*.
