# Supplementary material for: National Trends in Physical Activity Among Adults in South Korea Before and During the COVID-19 Pandemic, 2009-2021
Source: JAMA Netw Open. 2023 Jun 5;6(6):e2316930. doi: 10.1001/jamanetworkopen.2023.16930 (PMC10242425; doi:10.1001/jamanetworkopen.2023.16930)
Supplement: Supplement 1. — eFigure 1. Linear and Nonlinear Trends of Physical Activity for All Time Periods eFigure 2. Rate of Change in Prevalence of Physical Activity Before and After COVID-19 Pandemic Onset eTable. Results of a Systematic Rapid Review Reporting Trends of Physical Activity in Study Periods [file jamanetwopen-e2316930-s001.pdf]

Supplemental Online Content

Park S, Kim HJ, Kim S, et al. National trends in physical activity among adults in South Korea before and during the COVID-19 pandemic, 2009-2021. *JAMA Netw Open*. 2023;6(6):e2316930. doi:10.1001/jamanetworkopen.2023.16930

eFigure 1. Linear and Nonlinear Trends of Physical Activity for All Time Periods

eFigure 2. Rate of Change in Prevalence of Physical Activity Before and After COVID-19 Pandemic Onset

eTable. Results of a Systematic Rapid Review Reporting Trends of Physical Activity in Study Periods

This supplemental material has been provided by the authors to give readers additional information about their work.

© 2023 Park S et al. *JAMA Network Open.*

eFigure 1. Linear and Nonlinear Trends of Physical Activity for All Time Periods

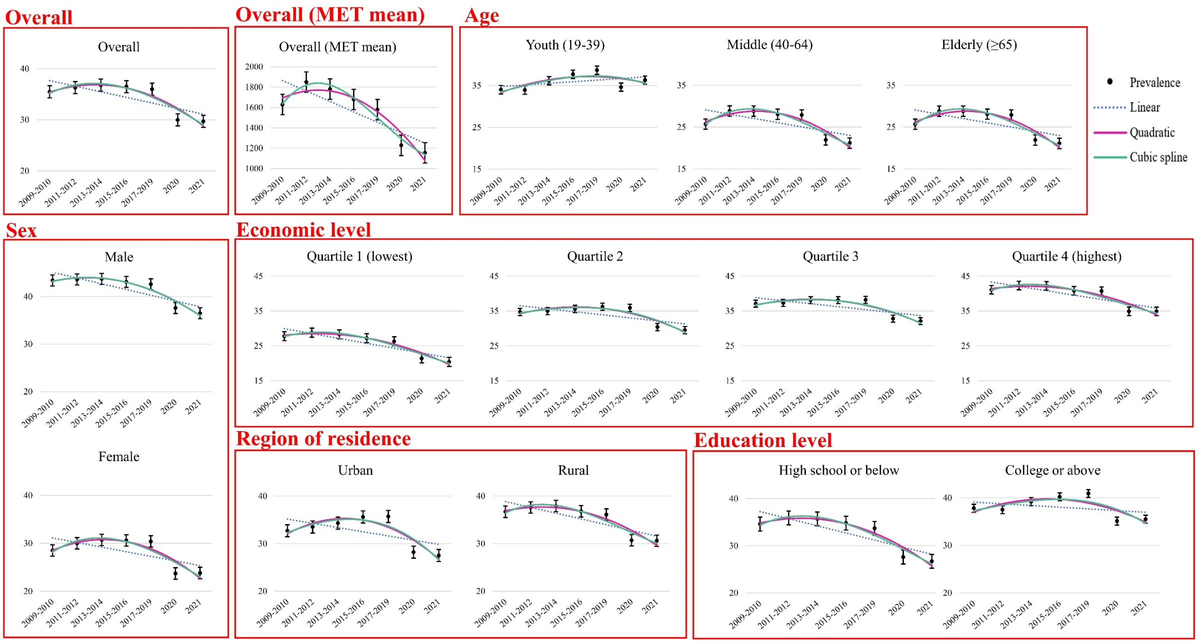

Abbreviations: MET, metabolic equivalent of task.

© 2023 Park S et al. *JAMA Network Open.*

eFigure 2. Rate of Change in Prevalence of Physical Activity Before and After COVID-19 Pandemic Onset

		Before				Intermediate	After	Physical activity trend, β		
		2009–2010 to 2011–2012	2011–2012 to 2013–2014	2013–2014 to 2015–2016	2015–2016 to 2017–2019	2017–2019 to 2020	2020 to 2021	Before pandemic	After pandemic	Trend difference
	Overall	2.25	1.38	-0.82	-1.37	-16.67	-1.00	1.0	-10.6	-11.6
Age, year	19–29	3.54	8.55	2.89	2.30	-8.73	6.83	14.3	-2.2	-16.6
	30–39	-2.93	5.14	5.17	2.46	-13.07	3.37	8.5	-6.5	-15.0
	40–49	-2.42	0.25	0.00	-1.48	-15.79	-0.89	-2.3	-10.7	-8.4
	50–64	3.23	-0.96	-2.91	-0.75	-16.37	-4.22	-2.4	-12.6	-10.2
	≥ 65	12.06	0.00	-2.43	-0.71	-21.51	-3.65	3.1	-13.4	-16.4
Sex	Male	0.46	0.23	-1.37	-1.16	-11.74	-2.93	-1.6	-9.3	-7.7
	Female	5.26	2.33	-0.33	-0.65	-22.04	0.42	4.3	-12.5	-16.8
BMI, kg/m^2	Underweight	9.42	4.51	-1.18	0.40	-17.79	0.00	7.5	-9.7	-17.1
	Normal	2.64	1.43	-0.85	-1.14	-17.82	-0.70	1.5	-11.1	-12.5
	Overweight	1.29	0.76	-2.27	-1.29	-15.18	-4.63	-1.6	-11.7	-10.0
	Obese	1.58	0.52	-0.52	-3.63	-16.67	1.94	-1.6	-9.5	-7.9
Region of residence	Urban	2.45	2.39	3.79	0.28	-21.01	-2.48	7.2	-14.1	-21.2
	Rural	2.45	0.80	-2.90	-1.90	-14.96	-0.33	-1.7	-9.2	-7.5
Educational level	High school or below	3.76	-0.56	-2.52	-3.16	-18.10	-3.26	-2.5	-12.1	-9.6
	College or above	-0.79	4.53	2.55	1.74	-14.18	1.14	7.5	-8.9	-16.3
Economic level	Quartile 1 (lowest)	3.60	-1.74	-3.89	-3.31	-18.63	-4.67	-4.7	-11.8	-7.0
	Quartile 2	0.86	1.71	1.69	-0.83	-15.32	-2.96	3.2	-10.5	-13.7
	Quartile 3	0.54	2.14	0.00	0.26	-14.14	-2.13	2.3	-9.6	-11.9
	Quartile 4 (highest)	2.92	-0.24	-3.32	-0.25	-14.25	0.00	-2.1	-9.3	-7.2
Smoking	Current smoker	1.22	0.48	-1.68	-1.95	-10.45	-4.17	-1.4	-8.8	-7.3
	Ex-smoker	0.47	-0.24	-1.89	-1.20	-12.41	-3.06	-2.8	-9.7	-6.9
	Never smoked	3.46	2.13	-0.30	-0.90	-19.28	0.37	3.0	-11.3	-14.3
Alcohol intake, days/month	< 1	4.03	0.97	-1.92	-0.33	-17.65	-1.59	1.1	-10.8	-11.9
	1–4	0.52	2.58	0.25	0.50	-12.50	1.43	3.4	-7.2	-10.6
	≥ 5	0.68	-1.12	-1.14	-2.07	-12.68	-2.15	-3.4	-9.4	-6.0
Diabetes	Yes	6.38	-0.95	-3.82	-0.33	-19.93	-2.90	-1.3	-12.7	-11.4
	No	2.23	1.63	-0.54	-1.08	-16.08	-0.97	1.7	-10.3	-12.0
Hypertension	Yes	6.13	0.91	-3.01	-3.42	-18.97	-2.78	-1.1	-12.1	-11.0
	No	1.91	1.34	0.26	-0.26	-15.61	-0.63	2.6	-10.1	-12.7
Depression	Yes	4.05	0.32	3.88	-4.05	-14.61	-4.18	3.2	-10.5	-13.7
	No	2.54	1.10	-0.82	-1.37	-16.67	-1.00	1.0	-10.6	-11.6
Stress status	Yes	6.39	-1.50	3.96	-1.47	-13.39	0.00	4.5	-7.9	-12.4
	No	2.54	1.10	-0.82	-1.37	-16.67	-1.00	0.9	-10.7	-11.6

Abbreviations: BMI, body mass index.

© 2023 Park S et al. *JAMA Network Open.*

10 **eTable.** Results of a Systematic Rapid Review Reporting Trends of Physical Activity in Study Periods

Author, year	Country	Samples	Subject (age; years)	Design	Collection type	Study period	Result	PA
**Include COVID-19 pandemic**								
Ajčević et al, 2021	Italy	400	Adult (>18)	C-S	Survey	2020.01–2020.03	During the lockdown, the number of steps dropped.	▼
Al-Musharaf et al, 2021	Saudi Arabia	297	Female (19-30)	C-S	Questionnaire	2019–2020	Since COVID-19, there has been an increase in weight related to increased actual total sleeping hours, decreased physical activity and coffee consumption.	▼
Cheval et al, 2021	France and Switzerland	377	Adult (mean age, 40)	C-S	Questionnaire, survey	2020.03–2020.04	With lockdown, more time was spent on walking and moderate physical activity. However, there were also adverse effects such as a decrease in vigorous physical activity and an increase in sedentary behavior.	–
Di Sebastiano et al, 2020	Canada	2,338	Adult (≥18)	C-S	App	2020.02–2020.04	MVPA and LPA significantly declined following the pandemic. Significant and sustained declines in LPA were observed even after 6 weeks of pandemic declaration.	▼
Ding et al, 2021	China	815	Adult (>18)	C-S	App	2019–2020	Step counts dropped sharply immediately after COVID-19 lockdowns, but soon began to increase. However, PA continues to decline due to COVID-19 lockdowns.	▼
Richardson et al, 2021	UK	117	Older adult (mean age, 76)	C-S	Survey	2020.03– 2020.05	Maintaining the level of PA before the lockdown after COVID-19.	–
Romero-Blanco et al, 2020	Spain	213	University student (mean age, 20.5)	C-S	Questionnaire	2020.01–2020.04	Since COVID-19, the PA and sedentary lifestyle of the participants has increased.	▲
Suzuki et al, 2020	Japan	165	Older adult (≥65)	C-S	Questionnaire	2017–2020	This study found that public health restrictions during the COVID-19 pandemic had a significant impact on the PA levels and subjective well-being of elderly individuals.	▼
To et al, 2021	Australia	60,560	Adult (18-79)	C-S	Program	2018–2020	There has been a decline in PA since the start of the COVID-19 lockdown, but it is quickly recovering.	▼
**Before COVID-19 pandemic**								

© 2023 Park S et al. *JAMA Network Open.*

Biernat et al, 2019	Poland	7,347	Adult (15-69)	C-S	Survey	2014–2018	Participation in LTPA and prevalence of recommended PA (≥600 MET-min/weeks) continued to increase. However, it is lower than in other Central and Eastern European countries.	▲
Du et al, 2019	US	27,343	Adult (≥18)	C-S	Survey	2007–2016	Adherence rates of the PAG did not improve over the study period, but sedentary time increased significantly over time.	▼
Guthold et al, 2018	Global (168 countries)	1.9M	Adult (≥18)	C-S	Questionnaire	2001–2016	Between 2001 and 2016, levels of insufficient PA were stable and over time, their prevalence increased in high-income countries than in low-income countries.	–
Griffin et al, 2020	Australia	107,171	Adult (18-75)	C-S	Survey	2004–2018	PA levels have increased since 2004, but there is considerable variation by employment status, with employed and inactive adults showing significant increases and unemployed adults showing no increase.	▲
Martins et al, 2019	Portuguese	9,856	Adolescent (11-17)	C-S	Survey	2002–2014	The prevalence of Portuguese girls meeting the PA guidelines (≥60MVPA-mins/day) is low, declines with age, and is stable from 2002-2014.	▼
Takamiya et al, 2019	Japan	286,704	Adolescent (>15, 1995–2011) and adult (>19, 2012–2016)	C-S	Survey	1995–2016	PA declined between 1995 to 2008, but the decline has since become unclear.	▼

Abbreviations: C-S, cross-sectional study; LPA, light physical activity; LTPA, leisure-time physical activity; MET, metabolic equivalent of task; MVPA, moderate-to-vigorous physical activity; PA, physical activity; PAG, physical activity guidelines for Americans.

© 2023 Park S et al. *JAMA Network Open.*
